# Profiles of bystanders' motivation to defend school bully victims from a self‐determination perspective

**DOI:** 10.1002/ab.21929

**Published:** 2020-08-30

**Authors:** Tomas Jungert, Kristoffer Holm, Nathalie O. Iotti, Claudio Longobardi

**Affiliations:** ^1^ Department of Psychology Lund University Lund Sweden; ^2^ Department of Psychology Turin University Turin Italy

**Keywords:** bullying, latent profile analysis, participant roles, person‐centered approach, student–teacher relationship

## Abstract

This study was aimed at exploring which latent profiles emerge based on ratings of self‐determined motivation to defend victims of bullying, and to explore if they are related to bystander roles and victimization in bullying, as well as student–teacher relations. Data were collected from 1,800 Swedish and Italian students, with an age range between 10 and 18 years (*M* = 12.6, standard deviation = 1.74). The students completed a survey in their classrooms. Latent profile analysis was used to explore the possible clusters of individuals with similar ratings on the motivational variables. Multivariate analysis of variances were conducted to explore differences between the profiles in relation to their roles when witnessing bullying and to student–teacher relationships. Four latent profiles emerged. The profiles represented respondents (a) high in prosocial motivation, (b) high in externally extrinsic motivation, (c) intermediate in externally extrinsic motivation, and (d) with identified/introjected motivation. Multivariate analyses showed that reports of bystander roles when witnessing bullying, teacher–student relationships, and bullying victimization, significantly differed over the motivational profiles. The bystanders were unevenly distributed across the four groups and most individuals were categorized in the prosocial motivation group. Female and male bystanders were evenly distributed across clusters. The prosocial motivation group experienced victimization to a lesser extent than the other profile groups. Students in the intermediate externally extrinsic group were more likely to take the pro‐bully and outsider role during bullying. Concerning student–teacher relationships, the prosocial motivation group reported the closest relationships with their teachers, while the intermediate externally extrinsic group reported the most conflictual relationships.

## 1 INTRODUCTION

During the last decades, aggressor and victim roles in school bullying situations have received considerable attention while bystanders, the individuals who witness the aggressions, have received significantly less attention from scholars. However, current research suggests that bystanders seem to have a major part in bullying (Denny et al., 2015; Polanin, Espelage, & Pigott, 2012; Pronk, Olthof, & Goossens, 2015; Saarento, Boulton, & Salmivalli, 2015). Some scholars highlight the complexity of bystanders as elucidated by the diversity of different roles that they seem to take, such as assistant, supporter, defender, and uninvolved/outsider (Demaray, Summers, Jenkins, & Becker, 2016; Pouwels, Lansu, & Cillessen, 2016; Salmivalli, Voeten, & Poskiparta, 2011). Defenders are the only actors that have the potential to stop and reduce bullying events. Bystanders are usually unmotivated to intervene if they have low defender self‐efficacy, fear being bullied themselves, the victim belongs to the outgroup, are morally disengaged from the bullying, think the bullying situation does not directly involve them, or believe the bullying is not severe (Cappadocia, Pepler, Cummings, & Craig, 2012; Jungert & Perrin, 2019; Thornberg & Jungert, 2014; Van Cleemput, Vandebosch, & Pabian, 2014). To become a defender, it is important to have a high level of motivation to defend (Jungert, Piroddi, & Thornberg, 2016). Researchers have begun to examine the bystander effect with children in school bullying situations (Jenkins & Nickerson, 2017; Machackova, Dedkova, & Mezulanikova, 2015). This study has found that girls are more likely to report defending than boys and younger students are more likely to report defending than older students (Fox, Jones, Stiff, & Sayers, 2014; Lambe, Hudson, Craig, & Pepler, 2017). According to self‐determination theory (SDT), individuals' motivation varies on a self‐volition continuum from amotivation (i.e., a lack of motivation) to intrinsic motivation (Deci & Ryan, 2000), with four distinct types in between: external, introjected, identified, and integrated regulation. External regulation involves motivation by tangible rewards or punishments. Introjected regulation refers to a form of partially internalized (i.e., internal but still outside those motivations, effects, and cognitions integrated with the self) motivation contingent on ego, pride, guilt, or shame. Identified regulation is the acceptance of the perceived value of a given behavior and involves the acceptance and personal valuing of an attained regulation. Compared with the former two, identified regulation is linked with greater commitment and performance in the given task (Deci & Ryan, 2008). Finally, integrated regulation involves not only accepting the importance of the behaviors but also fully integrating that importance with various aspects of the self.

Previous research has shown that identified and integrated regulations (often called autonomous motivation) predicts stronger persistence than external and introjected regulations (often called controlled motivation) in domains such as doing homework (Hagger et al., 2016), academic performance and learning (Niemiec & Ryan, 2009; Taylor et al., 2014), parenting satisfaction (Jungert et al., 2015), health behavior change (Ng et al., 2012; Ryan, Patrick, Deci, & Williams, 2008), work satisfaction (Van den Broeck, Lens, De Witte, & Van Coillie, 2013), and job performance (Moran, Diefendorff, Kim, & Liu, 2012), to name just a few. Moreover, possessing an integrated regulation to engage in prosocial behavior is linked with greater performance of such behavior when compared with external and introjected regulations (Hardy, Dollahite, Johnson, & Christensen, 2015). In the domain of school bullying, it has been found that warm student–teacher relationships are positively associated with intrinsic motivation to defend victims (Jungert et al., 2016). Still, extrinsic motivators can motivate children towards prosociality, particularly when they forge friendships with bullying victims (Bellmore, Ma, You, & Hughes, 2012), or receive tangible teacher approval for their actions (Thornberg et al., 2012),

In addition, some bystanders might combine various motives in a unique way, so that they, for instance, defend a victim both because they feel they should meet external demands and because they think it is important to help other people. Thus, different groups or types of bystanders might exist who may be categorized by different motivational profiles. When identifying motivational profiles, a person‐centered approach is required (Magnusson, 1998), which supplements the variable‐centered approach that is characteristically used in motivational research. The main goal in person‐centered analyses (e.g., cluster analyses) is to categorize individuals into groups whose members have similar motivational profiles, and it is expected to result in complementary information to the variable‐centered approach (Fortunato & Goldblatt, 2006). However, previous research by motivational scholars has not dedicated much attention to the person‐centered approach, particularly concerning school bullying. Our aims were (a) to map out the motivational profiles of bystanders to school bullying based on their scores for external, introjected, identified, and integrated regulation to defend victims of school bullying, as distinguished within SDT (Deci & Ryan, 2000; Jungert et al., 2016), and (b) to investigate how these different groups of bystanders differed on participant roles in bullying situations, teacher–student relations, and bullying victimization. We also compared age groups, gender, and two countries (Italy and Sweden) among the groups of bystanders.

### 1.1 Motivational profiles

Existing SDT‐based research has, with some recent exceptions, generally implemented a dimensional approach and has studied the unique effects of autonomous and controlled motivation through statistical methods such as regression analysis and path analysis (Ratelle, Guay, Vallerand, Larose, & Senécal, 2007; Ullrich‐French & Cox, 2009; Vansteenkiste, Sierens, Soenens, Luyckx, & Lens, 2009; Wang et al., 2017). The person‐centered approach in this study has several advantages, both theoretically and practically. For example, if individuals differ both in the quality and the quantity of their motivation (Ryan & Deci, 2017), it would be essential to find that some bystanders of school bullying recognized all four types of motives to defend victims, others scored low, and other combinations of them. Thus, we expected to find different clusters, each with a unique pattern of scores on motivation to defend (e.g., high on all types, low on all types, and high on some and low on some). A few person‐centered analyses of SDT's motivational constructs have been conducted, but most of them have focused on academic motivation (Ratelle et al., 2007; Ullrich‐French & Cox, 2009; Vansteenkiste et al., 2009). Yet, to date, no study has used a person‐centered analysis of SDT's motivational constructs in the domain of motivation to defend victims of bullying.

Furthermore, from a practical viewpoint, it is helpful to advance insights on the proportions of students characterized by an optimal or a suboptimal motivational profile. Gaining insight into students' motivational profiles may help schools develop motivational interventions that are tailored to each particular group. For instance, whereas some groups might benefit from autonomy support from teachers, other groups might require more structure, which can help to counteract pro‐bullying and passive bystander behavior by encouraging defender behavior. Efforts to influence bystander behavior are an important component of bullying prevention, such as in the KiVa program, which is designed to reduce negative bystander behavior and increase defender behavior (Kärnä et al., 2011; Salmivalli, Poskiparta, Ahtola, & Haataja, 2013). In the present study, the aim is to explore which latent profiles emerge based on ratings of self‐determined motivation and to explore whether they are differently related to bystander roles in school bullying, as well as student–teacher relations, and self‐reported victimization.

## 2 METHODS

### 2.1 Participants and procedure

A total of 1,800 boys (46%) and girls participated in the study. Eight participants (<1%) disclosed their gender as other. The participants were attending grades 4th to 12th in schools situated in the regions of Piedmont and Sardinia in Italy (*n* = 849) and in Southern Sweden (*n* = 951). A total of 29 schools and 100 school classes participated. The schools serve a low to middle‐class population. At the time of the study, the mean age of the sample was 12.6 years (standard deviation [*SD*] = 1.74; range: 10–18). All participants received school permission as well as active parental permission to participate before the collection of the data, along with giving their own consent to participate. None of the parents denied permission for their children to participate. The questionnaires were administered to the students during a class period. At least one researcher was present during data collection. The students had ∼20 min to complete the surveys. Participation was voluntary, and anonymity was guaranteed. The study was approved by the ethics board of the department at the Swedish University and by the ethical (institutional review board) committee at the Italian University (approval no. 118643).

Using parts of the same data set, two previous studies with other aims have been published (Iotti, Thornberg, Longobardi, & Jungert, 2020; Jungert et al., 2016).

### 2.2 Measures

#### 2.2.1 Motivation to defend

Motivation to defend was measured with the Motivation to Defend Scale. Italian and Swedish versions of this scale (Iotti et al., 2020; Jungert et al., 2016), which comprises 13 items based on SDT (Deci & Ryan, 2000), were used to assess the participants' motivation to defend victims during bullying episodes. Participants were asked to think of situations where they had witnessed another student being bullied and to report why they would help a victim. The scale consisted of four subscales measuring externally regulated extrinsic motivation (four items), introjected motivation (three items), identified motivation (three items), and intrinsic motivation (three items). Example items are “To be praised by a teacher” (extrinsic), “To avoid feeling guilty” (introjected), “Because I am the kind of kid who cares about others” (identified), and “Because I like to help other people” (intrinsic). Participants selected an answer that ranged from 1 (*Totally disagree*) to 5 (*Totally agree*). The internal consistency reliabilities for the subscales were *ω* = 0.67 (extrinsic), *ω* = 0.69 (introjected), *ω* = 0.63 (identified), and *ω* = 0.64 (intrinsic).

#### 2.2.2 Participant roles

Participant roles were measured with Italian and Swedish versions of a 15‐item self‐report scale (see Jungert et al., 2016; Thornberg & Jungert, 2013), which examined participants' tendency to fit several bullying‐related profiles during a school year—victim, bully, pro‐bully (who either actively assist bullies or cheer them on), outsider/passive bystander (not participating in bullying behavior but not stopping it), or defender (actively supporting victim). The items evaluating these profiles included “I tease some classmates, calling them nasty nicknames, threatening, or offending them” (bullying), “I laugh or cheer on the kids who tease or call a classmate nasty nicknames” (pro‐bullying), “When a classmate is hit or pushed, I stand by and I mind my own business” (passive bystanding), and “I defend classmates who are targeted by gossip or false rumors that are said behind their back” (defending). The participants indicated on a 5‐point scale how frequently they engaged in these behaviors in the last month, ranging from “it has never happened in the last month” to “more times a week.” Victimization was measured with a single‐item question, which also was referred to the current school year, “I have been bullied by classmates”, responded to with yes or no. One single‐item to measure bullying victimization has been considered reliable and sufficient when a construct is a concrete object that is effortlessly and uniformly imagined (Rossiter, 2002; Solberg & Olweus, 2003). Reliabilities were *ω* = 0.51 (bullying), *ω* = 0.69 (pro‐bullying), *ω* = 0.59 (passive bystanding), and *ω* = 0.81 (defending).

#### 2.2.3 Student–teacher relationships

Student–teacher relationships were measured using the Student Perception of Affective Relationship with Teacher Scale (SPARTS), a self‐report scale by Koomen and Jellesma (2015). The scale was used to measure the perception of the relationship with the main teacher of participants aged 9–14 years. Each item utilized a 5‐point scale ranging from 1 (no, i.e., not true) to 5 (yes, i.e., true). Factor analyses support the factorial validity of the SPARTS (Koomen & Jellesma, 2015). Reliabilities for the three subscales were *ω* = 0.78 (closeness), *ω* = 0.70 (emotional distance), and *ω* = 0.79 (conflictual).

### 2.3 Strategy of analysis

To explore possible clusters of individuals with similar ratings on the motivational variables, a latent profile analysis was conducted. The tidyLPA package (Rosenberg, Beymer, Anderson, & Schmidt, 2018), in R version 3.3.2 was used. The other analyses were performed in Jamovi v. 0.9.6.1 (Jamovi, 2019). Latent profile analysis is a type of finite mixture modeling that uses continuous variables as indicators of underlying (latent) clusters that group individuals who have responded similarly to items (i.e., belonging to the same profile). In this case, the four motivation variables, externally regulated extrinsic, introjected regulation, identified regulation, and intrinsic motivation were used to classify participants that have similar motivational profiles. The cluster solution is first constrained to two clusters, and subsequently, clusters are added while fit towards the data are evaluated until no further improvement is observed (Barnett et al., 2019). Measures used for evaluating data fit were primarily the Akaike information criterion (AIC), the Bayesian information criterion (BIC), sample size adjusted BIC (saBIC), and entropy values, as well as the bootstrap likelihood ratio test (BLRT; Berlin, Williams, & Parra, 2014; Celeux & Soromenho, 1996). Low AIC/BIC‐values represent better fit. Entropy, (range: 0–1), is a measure of the average accuracy of classifying an individual to a profile (Leiter & Maslach, 2016). Entropy values above 0.70 are considered acceptable (Jung & Wickrama, 2007). The BLRT compares the improvement in nested models, where a *p* < .05 indicates a statistically significant improvement with the addition of one more class (Berlin et al., 2014). The BLRT has been shown to be a very consistent indicator of the number of classes (Nylund, Asparouhov, & Muthén, 2007). As the profile solution is evaluated against fit to the data, the procedure is exploratory, revealing how many profiles best suit the available data.

To explore relationships with the cluster solution, the cluster classification was saved as a separate variable in the data set and used as a predictor in the subsequent analyses. To explore differences between the profiles in relation to their roles when witnessing bullying situations, and student–teacher relations, two separate multivariate analyses of variance (MANOVA) were conducted. In the first analysis, the cluster variable was entered as the independent variable, and the four participant roles (pro‐bullying, passive bystanding, defending, and bullying) were dependent variables. In the second analysis, the teacher relationships (closeness, emotional distance, and conflictual relations) were dependent variables. In the final analysis, we explored whether the cluster classification predicted bullying victimization, by the means of a logistic regression analysis. Cluster classification was entered as a predictor, and the dependent variable was the measure of bullying victimization (0 = no victimization and 1 = victimization). The profiles were dummy‐coded predictors in the regression model.

## 3 RESULTS

Table 1 displays descriptive statistics and intercorrelations between the study variables. Of the 1,389 participants that responded to the self‐report of bullying victimization, 342 (24.6%) reported that they had been subjected to bullying.

**Table 1:** Descriptive statistics and Pearson's correlations between the observed study variables

	EX	IJ	ID	IN	ProBullying	PASSIVE	DEF	BULLYING	CLOSE	NEXP	CF	Victimized
EX	–	−.01	−.18***	−.14***	.19***	.15***	−.1***	.1**	−.1**	.31***	.28***	−.08
IJ		–	.51***	.46***	−.07*	−.09**	.05	−.07	.05	.14**	.1	.07
ID			–	.62***	−.2***	−.22***	.13***	−.1**	.14***	−.07	−.06	.12***
IN				–	−.13***	−.21***	.25***	−.07	.22***	−.06	−.15***	.09
ProBullying					–	.39***	−.08**	.51***	−.2***	.14**	.18***	−.13***
PASSIVE						–	−.13***	.23***	−.16***	.07	.13***	−.04
DEF							–	−.14***	.31***	−.07	−.38***	.12***
BULLYING								–	−.13***	.16***	.1	−.26***
WARM									–	−.41***	−.4***	.08
NEXP										–	.59***	−.17***
CF											–	−.23***
Victimized												–
*M* (*SD*)	1.87 (−0.84)	3.73 (−0.94)	4.15 (−0.78)	3.83 (−0.83)	1.26 (−0.47)	1.84 (−0.86)	3.14 (−1.19)	1.17 (−0.35)	3.7 (−0.76)	1.73 (−0.59)	1.97 (−0.79)	–

Abbreviations: CF, conflictual relationships; CLOSE, closeness; DEF, defending; EX, extrinsic; ID, identified; IJ, introjected; IN, intrinsic; NEXP, negative expectations.

* *p* < .05.

** *p* < .01.

*** *p* < .001.

### 3.1 Latent profile analysis

We estimated models, starting with a 2‐profile model, and subsequently added profiles. As some of the fit indices improved, and others declined, we chiefly relied on the BLRT significance test for determining that improvements were significant with the addition of another profile. Significant improvements were observed up until four profiles. There were no significant improvements by adding a fifth or sixth profile. Therefore, the solution with four profiles was retained. The fit indices for the tested models are depicted in Table 2.

**Table 2:** Fit indices for the estimated latent motivation profiles (*N* = 1,800)

Profiles	Log‐likelihood	AIC	BIC	saBIC	Entropy	BLRT	*p*
2	−8007.45	16,052.9	16,157.32	16,096.96	0.84	395.80	<.01
3	−7887.63	15,823.3	15,955.16	15,878.91	0.85	239.64	<.01
4	−7877.07	15,812.2	15,971.52	15,879.39	0.81	21.12	<.01

Abbreviations: AIC, Akaike information criterion; BIC, Bayesian information criterion; BLRT, bootstrap likelihood ratio test; saBIC, sample size adjusted Bayesian information criterion.

**Figure 1:**
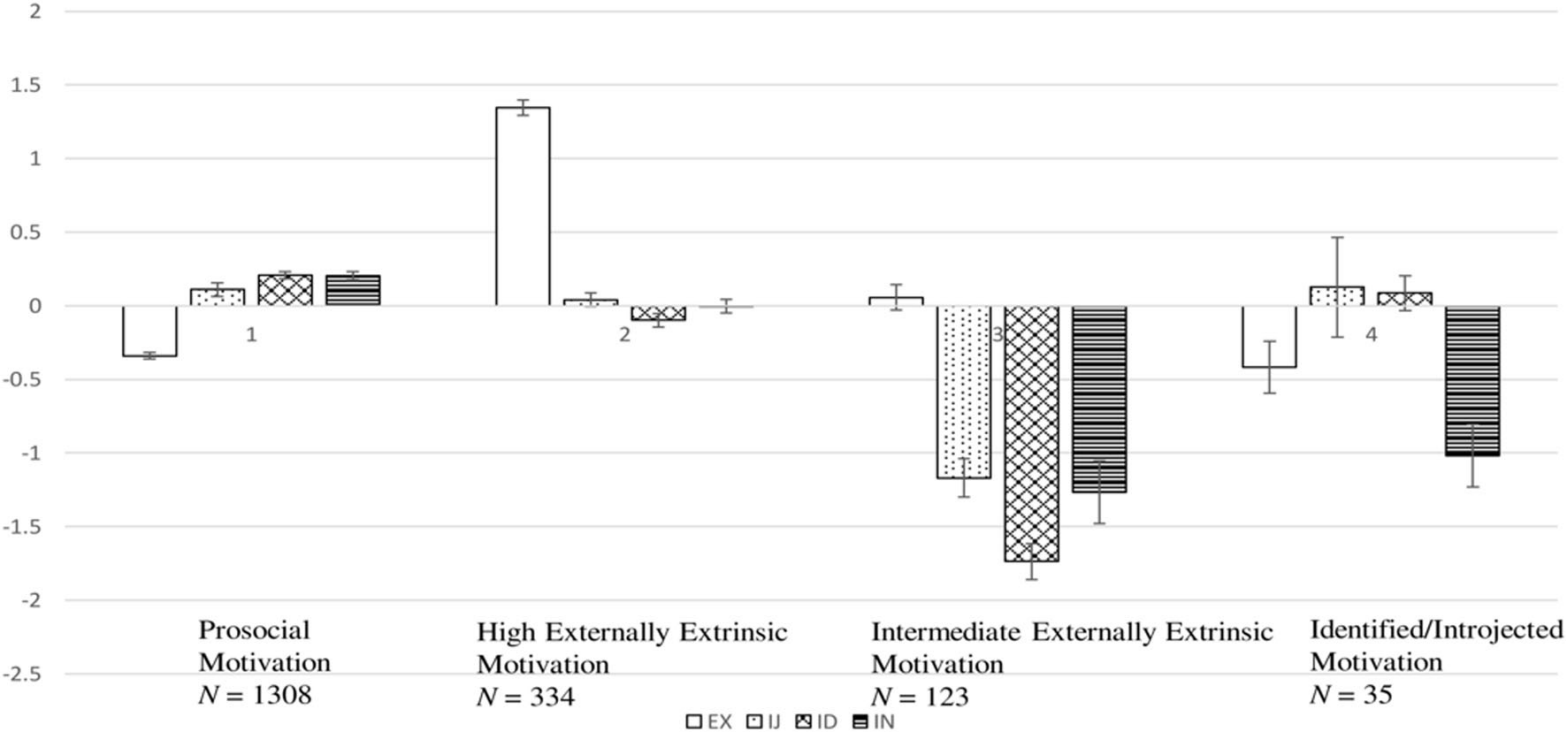
Latent profiles of self‐determined motivation among school children

The model that fit the data best consisted of four latent profiles. The four profiles contained 1,308 (72.7%), 334 (18.6%), 123 (6.8%), and 35 (1.9%), individuals respectively. The profiles are displayed in Figure 1. In comparison with the grand means of the motivation variables, as found in Table 1, it was clear that the largest profile group, (a) the prosocial motivation group, had higher ratings on the internal regulations introjected and identified motivation as well as intrinsic motivation, and lower ratings of externally regulated extrinsic motivation, than average. The ratings in the second largest group, (b) the high externally extrinsic motivation group (*n* = 334) were very similar to the grand means for introjected, identified, and intrinsic motivation, however, their ratings of externally regulated extrinsic motivation were notably higher. The third profile group, (c) the intermediate externally extrinsic motivation group, with 123 respondents was signified by lower ratings on all of the motivation variables apart from extrinsic, which was slightly higher than average. The last, smallest profile, (d) the identified/introjected group (*n* = 35), was lower in both externally regulated extrinsic and intrinsic motivation but had higher levels of introjected and identified regulations. The profiles were not significantly related to gender *χ* ^2^(3) = 2.36, *p* = .50. However, there was a significant association between profile and country, *χ* ^2^(3) = 28.64, *p* < .001, where Swedish students were more likely to belong to the high externally extrinsic motivation group, and Italian students were more likely to belong to the identified/introjected group. A one‐way analysis of variance showed a significant association with age *F*(3, 134.14) *p* < .001. A Tukey post hoc test showed that participants in the identified/introjected group (*M* = 12.21, *SD* = 1.59) were significantly younger than those in the intermediate externally extrinsic motivation group (*M* = 12.81, *SD* = 1.87, *p* < .01) and the high externally extrinsic motivation group (*M* = 12.61, *SD* = 1.76, *p* < .01).

### 3.2 Multivariate analyses

In the next step, we investigated the relationship between profiles and participant roles, teacher relations, and bullying victimization. A MANOVA with the four participant roles as dependent variables revealed a significant main effect of profiles, Pillai's trace = 0.077, *F*(12, 2,232) = 4.88, *p* < .001, partial *η* ^2^ = 0.026. After the overall multivariate test was significant, we further explored univariate main effects. Bonferroni‐corrected one‐way ANOVA for each dependent variable, with Tukey post hoc tests were conducted to investigate the differences, which revealed that significant differences could be found within all the variables. For pro‐bullying, the intermediate externally extrinsic motivation group and the prosocial motivation group differed significantly (*p* < .001), where those in the intermediate externally extrinsic profile scored significantly higher on pro‐bullying. In the case of passive bystanding, the intermediate externally extrinsic motivation profile and the prosocial profile significantly differed (*p* < .001), with higher mean levels for those with intermediate externally extrinsic profiles. There was also a significant difference between the intermediate externally extrinsic motivation profile and the high externally extrinsic motivation profile (*p* = .046), and the prosocial profile and high externally extrinsic motivation profile (*p* = .031), where the mean levels were higher for the intermediate externally extrinsic profile than the high externally extrinsic profile, and the prosocial profile reported lower mean levels than the high externally extrinsic profile. For defending, the intermediate externally extrinsic motivation group and the prosocial profile differed significantly (*p* = .001) as those in the prosocial profile reported higher levels of defending. The prosocial profile and the high externally extrinsic motivation profile also significantly differed (*p* < .001), where the prosocial profile reported higher mean levels of defending. For bullying, only the intermediate externally extrinsic motivation profile and the prosocial profile significantly differed (*p* = .039), where the intermediate externally extrinsic had higher mean levels. The means are displayed in Table 3.

**Table 3:** Means and standard deviations of study variables for each latent profile

	Profile	Pro‐Bullying	Passive	Defending	Bullying	Close	NEXP	CF
Mean (*SD*)	Low motivation	1.55 (−0.59)	2.44 (−1.03)	2.92 (−1.2)	1.27 (−0.44)	3.33 (−0.87)	1.84 (−0.73)	2.1 (−0.78)
	Intermediate motivation	1.21 (−0.4)	1.95 (−0.8)	2.68 (−1.15)	1.12 (−0.2)	3.47 (−0.73)	1.54 (−0.31)	2.34 (−1.0)
	Prosocial motivation	1.21 (−0.4)	1.74 (−0.8)	3.22 (−1.2)	1.14 (−0.3)	3.77 (−0.75)	1.65 (−0.55)	1.87 (−0.79)
	High motivation	1.37 (−0.6)	1.99 (−0.95)	3.00 (−1.14)	1.21 (−0.44)	3.65 (−0.69)	2.03 (−0.64)	2.22 (−0.72)

Abbreviations: CF, conflictual relationships; NEXP, negative expectations; *SD*, standard deviation.

Next, a MANOVA was conducted with the teacher relations as dependent variables. The results showed that there was a significant main effect of profiles. Pillai's trace = 0.11, *F*(9, 1,479) = 6.49, *p* < .001, partial *η* ^2^ = 0.038. Univariate analyses with Tukey post hoc tests demonstrated that there were significant differences between the intermediate externally extrinsic motivation profile and the prosocial profile (*p* < .001), as well as the intermediate externally extrinsic motivation profile and the high externally extrinsic motivation profile (*p* = .002), for the close relations variable. In these comparisons, the mean levels were the highest for the prosocial profile, followed by the high externally extrinsic, and lowest for the intermediate externally extrinsic. For emotional distance, the prosocial motivation profile and the high externally extrinsic motivation profile differed significantly (*p* < .001), with higher mean levels being reported by those in the high externally extrinsic profile. Lastly, for conflictual relations, the intermediate externally extrinsic motivation group and the prosocial motivation profile differed (*p* = .005), as well did the prosocial profile and the high externally extrinsic motivation profile (*p* = .005). Here, the mean levels for the intermediate externally extrinsic profile and the high externally extrinsic profile were both higher than those reported by the prosocial profile. See Table 3 for the means and standard deviations split by profiles.

In the final analysis, a logistic regression model was estimated, with profiles predicting bullying victimization. The largest profile (the prosocial motivation profile) was used as the reference category. The overall model was significant *χ* ^2^(3) = 14.65, *p* = .002, Nagelkerke's *R* ^2^ = .016. The results showed that two of the three other profiles significantly predicted reports of bullying victimization, odds ratio (OR)_intermediate externally extrinsic motivation profile_ = 1.99, 95% confidence interval (CI) [1.31, 3.01] *p* < .001, OR_identified/introjected motivation profile_ =1.79, 95% CI [0.75, 4.23] *p* = .19, and OR_high externally extrinsic motivation profile_ = 1.46, 95% CI [1.08, 1.97] *p* = .013. The OR of each predictor should be interpreted as the relative increase in odds of reporting having been bullied, in comparison with the reference category. Although the estimate for the identified/introjected motivation profile also appeared to be large, the confidence interval demonstrated that the standard error (*SE*) for this estimate was relatively large (*SE* = 0.44). This is not surprising, due to the very small amount of participants in this profile.

## 4 DISCUSSION

The aim of the study was both to extend the limited amount of research on motivational profiles of students from an SDT perspective and to deepen the knowledge about bystanders' motivation to defend victims. We investigated the motivational dimensions of bystanders who witness school bullying and examined the utility of this approach for understanding and explaining students’ experiences. Four distinct combinations of motivation regulations emerged from the analyses.

Indication of a stable four‐cluster solution was found reflecting (a) the prosocial motivation group, (b) the high externally extrinsic motivation group, (c) the intermediate externally extrinsic group, and (d) the identified/introjected regulations group. The results included both similarities and differences in experiences among the four profiles. The results are related to the type and size of the motivational profiles, their relation with student–teacher relationships, their relation to experiences of taking various participating roles in school bullying situations and of being victimized, and differences between age groups, and country (Italy and Sweden). These four clusters signified the most parsimonious and interpretable cluster solution. Consistent with prior studies based on SDT, the clusters show that autonomous and controlled motivation are comparatively orthogonal constructs. However, the distribution of bystanders across the four groups was uneven, varying from 2% to 73%. Most individuals were categorized in the prosocial motivation group, and only a very small percentage of the bystanders belonged to the identified/introjected group.

In contrast to gender differences in motivational dimensions that have been found in academic motivation (Ratelle et al., 2007; Vansteenkiste et al., 2009; Vansteenkiste, Zhou, Lens, & Soenens, 2005), female and male bystanders were relatively evenly distributed across the clusters. Previous research has found that females tend to be overrepresented in motivation groups characterized by autonomous regulation and less likely to belong to controlled motivation groups. Regarding prosocial motivation in school bullying situations, said gender difference does not seem to exist. In addition, in line with previous studies, we found an association with respect to age groups in the clusters, where those belonging to the high externally extrinsic motivation group were relatively younger than those in the intermediate externally extrinsic group. Earlier research has found a gradual deterioration of intrinsic academic motivation across the school years (Gottfried, Fleming, & Gottfried, 2001; Vansteenkiste et al., 2009), whereby younger school students have been more likely to belong to motivation groups characterized by high intrinsic and identified motivation and less likely to belong to groups high in externally regulated extrinsic motivation and low in intrinsic and identified motivation, while the reverse was true for older students. Such patterns also seem to exist when it comes to motivation to defend victims of school bullying. In relation to this, it has been found that helpful bystander behaviors are likely to decline with age (Endresen & Olweus, 2001; Rogers & Tisak, 1996). The only age group that differed substantially from the others was 14‐year olds. They were overrepresented in the intermediate externally extrinsic group and had a lower representation in the prosocial motivation group. This could be related to findings indicating that bullying peaks around the age of 14 and that students in that age group have stronger pro‐bullying attitudes (Salmivalli & Voeten, 2004). Our results could possibly explain previous findings that younger students are more likely to defend (Endresen & Olweus, 2001; Lambe et al., 2017; Rogers & Tisak, 1996) by showing that they may have motivation profiles that are more autonomously regulated than 14 year olds. However, we also found that students older than 14 years also have profiles that are more autonomously related, so it is likely that prosocial motivation to defend increases as students get older than 14 years.

Few differences between Italian and Swedish students were found. This could have been expected as the countries display few differences in the cultural dimensions as reported by Hofstede (2001) and both countries show similar levels of quality of life (per capita gross domestic product), education indices, infant mortality, life expectancy, and literacy rates (UNICEF, 2016). Surprisingly, Swedish students were more likely to belong to the high externally extrinsic group while Italian students were more likely to belong to the identified/introjected group. It is possible Italian bystanders have stronger attitudes against bullying, which was found in a previous study that compared children from Italy and Singapore (Pozzoli, Ang, & Gini, 2012). In line with SDT (Ryan & Deci, 2017), Italian students might have internalized their antibullying attitudes and prosocial values to a higher extent than the Swedish students, which made Italians more likely to belong to the identified/introjected group and Swedes to belong to the externally extrinsic group.

### 4.1 Associations with being victimized and participants roles

The prosocial motivation group experienced school bullying victimization to a lesser extent than the other profile groups. This finding is in line with a previous study that found that prosocial children were significantly more popular than bullies, victims, and bully victims (Warden & Mackinnon, 2003). As suggested by Warden and Mackinnon (2003), prosocial children may be better at constructively resolving interpersonal uncertainties than other children, which may protect them from being victimized. Less prosocial children are more likely to be rejected and thus risk becoming victims of school bullying.

Students in the intermediate externally extrinsic group took the outsider role more often than the high externally extrinsic motivation group and the prosocial motivation group, while the latter group took the role of outsider even less often than the high externally extrinsic motivation group. The same pattern emerged regarding the pro‐bully role, where the intermediate externally extrinsic group was most prone and the prosocial motivation group was least likely to take the role as a pro‐bully. These findings confirm SDT's claim that autonomously motivated students would be less pro‐bully directed and less passive when witnessing school bullying, while bystanders more controlled in their prosocial motivation are more likely to take on a passive and a pro‐bully role (Jungert et al., 2016).

### 4.2 Associations with student–teacher relationships

Overall, the prosocial motivation group reported the closest relations with their teachers, which supports findings by Jungert et al. (2016) who also found that the prosocially motivated young adolescents experienced their relationships with the teacher as closer than other adolescents did. Furthermore, the high externally extrinsic motivation group had student–teacher relationships characterized by the highest emotional distance. Since negative expectations (i.e., emotional distance), largely are believed to be a negative relational factor (Koomen & Jellesma, 2015), this type of student–teacher relationship fails to meet the basic needs of autonomy and relatedness, which has been associated with controlled types of motivation. As the high externally extrinsic motivation group was higher in externally regulated extrinsic motivation than the other groups, it makes sense that they also had more emotional distance towards their teachers. In addition, more insecure students might feel that the teacher should attend more to their needs, while also experiencing the introjected motivators of guilt or shame (Deci & Ryan, 2000). This was distinctive for the identified/introjected group. In addition, the intermediate externally extrinsic group had higher levels of conflictual relationships with their teachers than the prosocial profile, and the least close relationships. This is in line with previous research showing conflictual student–teacher relationships to be associated with aggressive and externalizing behaviors (Marengo et al., 2018; Roorda, Verschueren, Vancraeyveldt, Van Craeyevelt, & Colpin, 2014), less prosocial behavior (Birch & Ladd, 1998), and extrinsic motivation to defend victims of bullying (Jungert et al., 2016).

### 4.3 Limitations and future research directions

The current study has some limitations, including the self‐report assessment, which might artificially increase the observed strength of the relationships between variables through shared method variance. Such problems could be evaded by comprising teacher reports on their students' participant roles. In addition, the research was cross‐sectional, preventing the inference of causal relationships. Longitudinal research in combination with cross‐lagged analyses is needed to sort out whether more autonomously motivated bystanders actually defend victims of school bullying and if being a victim oneself predicts belonging to the inferior quality motivation groups or whether autonomously motivated bystanders experience less victimization than students in the other groups. Furthermore, longitudinal analyses would allow exploration of whether the bystander groups would show different motivational paths over time and whether some students might change to a different cluster because of being exposed to a particular bullying situation. However, a solution to the issue would be to conduct experiments, where a possibility is to conduct randomized impact evaluations of interventions. For example, one could evaluate whether an intervention to promote better student–teacher relationships (i.e., closer, less emotionally distant and conflictual) lead to increases in prosocial motivation to defend victims and/or to reductions in externally extrinsic motivation to defend.

Another limitation was the use of a single item to measure self‐reported victimization, as this can be sensitive to measurement error. Yet, single‐item measures may be acceptable if the item represents a homogenous and unidimensional construct (Wanous & Hudy, 2001).

It should also be noted that the reliabilities for some of the subscales were in the lower range, such as intrinsic motivation, passive bystanding, and notably, the bullying variable. It is possible that the low reliabilities of these scales were a consequence of the relatively few amount of items used to assess them (3 per subscale), and in the cases of passive bystanding and bullying, a combination of few items and non‐normal distributions, as most respondents reported low levels of having bullied or passively watching bullying take place. Non‐normal data has a tendency to reduce reliability estimates (Zhang & Yuan, 2016).

Finally, we referred only to traditional bullying and not cyberbullying, which is a very common phenomenon among adolescents and preadolescents. Thus, it is unclear whether the same motivational profiles would have arisen, had we also included cyberbullying in the study. The results should, therefore, be interpreted with caution.

Future research could include measures of amotivation. There are two forms of amotivation; one is about believing that acting will not yield a desired outcome, while the other is about believing that one cannot perform adequately (Pelletier, Dion, Tuson, & Green‐Demers, 1999). In school bullying, both of these types may be adequate. Thus, future research could examine if amotivation is part of an additional cluster variable or if a fifth cluster would emerge. In addition, a strength of the present study was the large sample size, which allowed for identification of smaller clusters, like the one that consisted of merely 2% of the participants. However, future studies should attempt to replicate these profiles in different samples to investigate whether they consistently emerge.

## 5 CONCLUSION

In conclusion, we identified four profiles with significant differences amongst themselves. The general tendency we found in these profiles advances our knowledge besides what we already know from variable‐centered studies. For instance, students can have a combination of both autonomous and controlled regulations as well as high and low‐quality motivation that is related to less favorable outcomes. It is not only the absence of motivation that is detrimental but also the presence of certain types of regulation is associated with negative outcomes, even if the students have average levels of prosocial motivation.

Overall, these findings advance our knowledge of how bystander motivation and participant roles work and might help to improve the effectiveness of future antibullying interventions, given that researchers and professionals might employ the knowledge acquired from cluster analyses to devise programs that are better tailored to fit students’ individual needs and tendencies. One suggestion for teachers might be to try to promote more positive relationships within the entire class, where possible, as this can be a protective factor for bullying (Longobardi, Iotti, Jungert, & Settanni, 2018; Thornberg, Wänström, Hong, & Espelage, 2017). Another suggestion is to encourage perspective taking among students, as that could help with fostering prosocial motivation (Roth, Kanat‐Maymon, & Bibi, 2011). Finally, a subject that can sometimes be difficult for students to embrace is class/school rules, as they can be seen as impositions from the outside. However, providing meaningful rationales for said rules and explaining the consequences of breaking them, hence, creating a form of interpersonal agreement between students can help them embrace the rules in a more autonomous manner. This might help students to adopt more prosocial motivation profiles.

## CONFLICT OF INTERESTS

The authors declare that there are no conflict of interests.

## Data Availability

The data that support the findings of this study are available from the corresponding author upon reasonable request
